# Synthesis, characterization, and molecular modeling of phenylenediamine-phenylhydrazine-formaldehyde terpolymer (PPHF) as potent anti-inflammatory agent

**DOI:** 10.1016/j.heliyon.2023.e18067

**Published:** 2023-07-06

**Authors:** N. Mujafarkani, Victoria Bassey, Jumbo J. Tokono, A. Jafar Ahamed, Innocent Benjamin, Daniel C. Agurokpon, Yohanna J. Waliya, Hitler Louis

**Affiliations:** a PG and Research Department of Chemistry, Jamal Mohamed College (Autonomous), (Affiliated to Bharathidasan University), Tiruchirappalli, 620020, Tamilnadu, India; b Computational and Bio-Simulation Research Group, University of Calabar, Calabar, Nigeria; c Department of Pure and Applied Chemistry, University of Calabar, Calabar, Nigeria; d Department of Microbiology, University of Cross River State, Calabar Nigeria; e Faculty of Allied Health Sciences, Chettinad Hospital and Research Institute, Chettinad Academy of Research and Education, Kelambakkam 603103, Tamil Nadu, India

**Keywords:** Terpolymer, Synthesis, Characterization, DFT, Inflammatory, Molecular docking

## Abstract

Inflammation, a characteristic physiological response to infections and tissue damage, commences with processes involving tissue repair and pathogen elimination, contributing to the restoration of homeostasis at affected sites. Hence, this study presents a comprehensive analysis addressing diverse aspects associated with this phenomenon. The investigation encompasses the synthesis, spectral characterizations (FT-IR, ^1^H NMR, and ^13^C NMR), and molecular modeling of p-phenylenediamine-phenylhydrazine-formaldehyde terpolymer (PPHF), a potent agent in promoting inflammation. To explore the reactivity, bonding nature, and spectroscopy, as well as perform molecular docking for in-silico biological evaluation, density functional theory (DFT) utilizing the def2svp/B3LYP-D3BJ method was employed. The results reveal significant biological activity of the tested compound in relation to anti-inflammatory proteins, specifically 6JD8, 5TKB, and 4CYF. Notably, upon interaction between PPHF and 6JD8, a binding affinity of −4.5 kcal/mol was observed. Likewise, the interaction with 5TKB demonstrated an affinity of −7.8 kcal/mol. Furthermore, a bonding affinity of −8.1 kcal/mol was observed for the interaction with 4CYF. Importantly, these values closely correspond to those obtained from the interaction between the proteins and the standard drug ibuprofen (IBF), which exhibited binding affinities of −5.9 kcal/mol, −7.0 kcal/mol, and −6.1 kcal/mol, respectively. Thus, these results provide compelling evidence affirming the tremendous potential of p-phenylenediamine-phenylhydrazine-formaldehyde (PPHF) as a highly promising anti-inflammatory agent, owing to the presence of nitrogen—a heteroatom within the compound.

## 1 Introduction

**Scheme-1:**
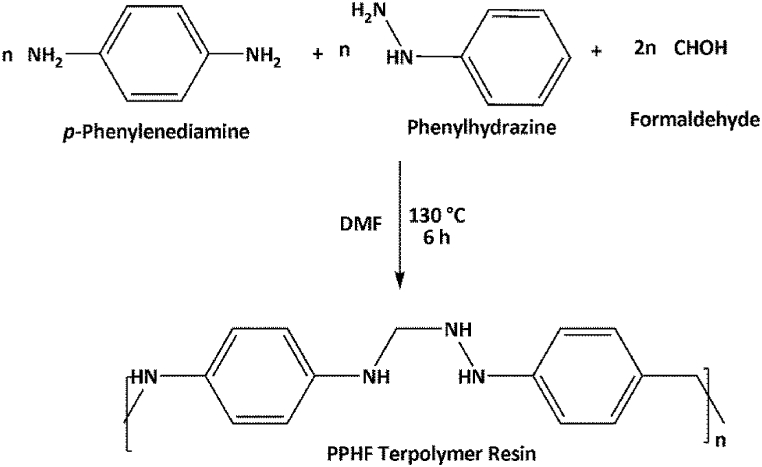
Synthesis Route of PPHF Terpolymer Resin.

Inflammation is a symptom of an inflammatory disease, which is characterized by an overactive immune system that targets healthy tissues [1]. The immune system of the body typically responds to damage, infection, or irritation by causing inflammation. In contrast, the immune system wrongly views healthy tissues as foreign invaders in inflammatory illnesses and begins to attack them [2]. There are many different types of inflammatory diseases, such as: Rheumatoid arthritis which is a persistent autoimmune condition that affects the joints and results in swelling, discomfort, and stiffness [3]. Psoriasis which is a persistent skin disorder results in the formation of red, scaly skin patches as a result of inflammation [4]. Multiple sclerosis which involves a long-term autoimmune condition that damages the myelin sheath that covers the central nervous system and produces inflammation [5]**.** Inflammatory diseases can affect the joints, skin, gut, lungs, kidneys, blood vessels, and brain, among other bodily components [6] which are lethal to the human health conditions (see Scheme 1).

More so, inflammatory disorders, whose exact origins remain unknown, are believed to arise from a combination of genetic, environmental, and lifestyle factors [7]. These disorders can manifest as acute or chronic inflammation, with the latter potentially causing permanent damage to affected tissues or organs if left untreated [8]. The impact of inflammatory disorders on an individual's quality of life can be substantial, leading to pain, fatigue, disability, and emotional distress [9]. Certain populations or age groups may be more prone to specific inflammatory disorders [10]. For instance, rheumatoid arthritis is more prevalent among women and older individuals, while inflammatory bowel disease is commonly observed in younger adults [11]. The treatment of inflammatory diseases typically involves the use of nonsteroidal anti-inflammatory drugs (NSAIDs), corticosteroids, disease-modifying antirheumatic drugs (DMARDs), biologic agents, immunosuppressants, as well as dietary and lifestyle modifications such as exercise, stress reduction, and smoking cessation [12]. While inflammatory diseases cannot be cured, effective management strategies exist [13].

Due to the increase in the rate of inflammatory disease and the reduced efficacy of the available antibiotics. Studies have attempted to develop the therapeutic agents to improve the treatment of various types of diseases. For example, Refat and Fadda studied the antibacterial activities of Isoxazole, Pyrazole, Pyrimidine Derivatives and Triazine as Novel Hydrazide [14]. The available closely related literatures at our disposal could give us only infinitesimal insight into this study. Therefore, some studies related to anti-inflammation used Ibuprofen- Tranexamic Acid Codrug [15], New Diclofenac Derivatives [16], pyrazolopyrimidine core [17], Heterocyclic Analogues of 2-Substituted Benzimidazole [18], substituted 4, 5-dihydro-2H-indazole derivatives [19], pyridinylpyrazole and pyridinylisoxazole derivatives [20] based on the challenges mentioned above. There is a need to develop new therapeutic agent that will perform considerably better than the available treatment options. Thus, this study was designed to investigate the synthesis, characterization and molecular modeling of phenylenediamine-phenylhydrazine-formaldehyde terpolymer (PPHF) as effective inflammatory agent.

Herein, we determine the biological potency of *p*-phenylenediamine-phenylhydrazine-formaldehyde terpolymer (PPHF) as an anti-inflammatory agent. The theoretical calculations were carried out using the Density function theory at the B3LYP/6–31 G (d, p) level of theory. The experimental and theoretical frequencies were compared with the FT-IR analysis. The Frontier molecular orbital analysis which consists of the HOMO and the LUMO and the corresponding global quantum descriptors such as the global softness and hardness, chemical potential, electronegativity and Electrophilicity index were calculated at the same level of theory. In order to further elucidate on the charge transfer and stability of the studied compound, the Natural bond orbital analysis based on the second order perturbation theory was analyzed. Furthermore, to provide an insight with the biological potency of the studied compound, molecular docking was also carried out against three inflammatory protein 6JD8, 4CYF and 5TKB. A commercial drug ibuprofen was equally used as a standard so as to measure the efficacy of the studied compound.

## 2 Methods

### 2.1 Experimental studies

#### 2.1.1 Synthesis of terpolymer “PPHF”

A mixture of *p*-phenylenediamine (1 mol) and phenyl hydrazine (1 mol) with formaldehyde (2 mol) were dissolved in dimethylformamide as a reaction medium and mixture was taken in a round bottom flask. They were refluxed in the oil bath at 130 °C for 6 h. The content of the flask was periodically shaken well to ensure homogeneous mixing. After the stipulated reaction time, the content of the flask was poured into a beaker containing ice crystals with vigorous shaking and left overnight. The separated resin was washed with warm water. The resin sample was air-dried and extracted with ether to remove untreated monomers.

The obtained resin was purified twice by dissolving in 1:1 (HCl/water) and regenerating by addition of 12% NaOH with constant stirring. The resin was filtered off, washed with hot water and methanol, acetone and air dried at 75 °C for overnight in an air oven. The dried sample was finely grinded and sieved to obtained uniform particle of 100 mesh size and stored in a polyethylene container. Then the synthesized resin was used for further characterization.

#### 2.1.2 Gel permeation chromatography

One of the widely used methods for the routine determination of molecular weight and molecular weight distribution is gel-permeation chromatography (GPC), which employs the principle of size-exclusion chromatography to separate samples of polydisperse polymers into fractions of narrower molecular-weight distribution.

#### 2.1.3 Spectral characteristics

The FTIR spectral study is widely employed by several researchers to characterize terpolymer various types. IR Spectroscopy has been widely used to identify the existence of functional groups in the synthesized compound Shimadzu (model) whilst FTIR is being used to locate the functional groups present in terpolymer resin.

The NMR spectra of the terpolymer resin were recorded in DMSO‑*d*_6_ solvent using Bruker 400 MHz. The ^1^H NMR spectrum was recorded to identify the protons present in the terpolymer and into account of the peak positions and its intensity. The ^13^C NMR spectrum provides the information about the nature of the carbon skeleton present in the resin.

The terpolymer sample obtained from *p*-Phenylenediamine and phenylhydrazine with formaldehyde was dark brown colour in which dimethylformamide was used as a reaction medium. Solubility behaviours of the terpolymer sample have been studied in various solvents. It was discovered that the terpolymer was soluble in dimethylsulphoxide (C₂H₆OS), HCl, H₂SO₄ but it was insoluble in other solvents like chloroform, benzene, toluene, ethanol, and ether. Based on the analytical data, the empirical formula of the repeating unit is found to be C_16_H_22_N_4_, which is in good agreement with the calculated value of C, H, and N (see 3.0).

The NMR was scanned at 400 MHz with dimethylsulphoxide (DMSO) solvent. The signal appeared at 6.6–7.6 (*δ*) ppm is assigned to all the protons of aromatic ring. The signal showed at 10.4 (*δ*) ppm is assigned to the –NH bridge in the terpolymeric ligand. The signal observed at 4.8 (*δ*) ppm is assigned to the Ar-N-CH_2_ linkages. The signal appeared at 2.5 (*δ*) ppm is assigned to the methylene group in the terpolymer. ^13^C NMR spectrum shows the corresponding peaks at 118.81, 112.36, 113.98, 119.17, 115.16 and 115.89 (*δ*) ppm with respect to C_1_–C_6_ of the aromatic ring of *p*-phenylenediamine. The peak appeared at 129.41, 128.80, 119.37, 129.28, 129.98 and 137.33 (*δ*) ppm, is assigned to the C_1_–C_6_ of the aromatic ring of phenyl hydrazine. The peak appeared at 40.58 (*δ*) ppm is assigned to the –CH_2_ group in the terpolymer. The peak appeared at 145.23 (*δ*) ppm is due to the C–N group.

### 2.2 Computational details

The Becke-3-Parameter-hybrid model of Lee-Yang-Parr (B3LYP) correlation function along with D3BJ empirical dispersion embedded in the Gaussian 09 package [21] was used in theoretical studies based on density functional theory (DFT) theory. This functional is one of the mostly used hybrid functional. The molecular geometries that led to energy minima were optimized using the same parameters so as to minimize the energy of the studies compound and reduce the inter molecular forces to zero state. The HOMO-LUMO energies were calculated at the same level of theory based on the Koopmans theorem. Additionally, the quantum calculations were carried out using the following equations:

(1) Energy gap = E_LUMO_ – E_HOMO_

(2) η = − 1/2(E_HOMO_ − E_LUMO_)

(3) σ = 1/η

(4) μ = − 1/2(E_HOMO_ + E_LUMO_)

(5) χ = 1/2(E_HOMO_ + E_LUMO_)

(6) ω = μ2 / 2η

HOMO-LUMO plot was obtained using the Chemcraft application software. The intermolecular and intramolecular charge transfer was examined using the NBO analysis whereas the molecular electrostatic potential analysis plot of the studied compound was obtained and used to determine the positive, negative and neutral region of the studied compound.

### 2.3 Molecular docking protocol

To assess the biological activity of the studied compound, molecular docking was performed using Autodock Vina 4.2 [22], and the resulting interactions were visualized using Biovia Discovery Studio software [23]. The protein structures relevant to inflammatory disease, namely 6JD8 [24], 5TKB [25], and 4CYF [26], were obtained in pdb format from the Research Collaboratory for Structural Bioinformatics Protein Data Bank (RCSB PDB) [27]. Prior to interaction analysis, these target proteins underwent preparation using Biovia Discovery Studio software, involving the removal of water molecules, heteroatoms, and ligands. Additionally, a comparative study was conducted by docking a standard drug, ibuprofen (IBF), which is commonly used for the treatment of inflammatory disease, with these proteins.

## 3 Results and discussion

The terpolymer sample obtained from *p*-Phenylenediamine and Phenyl hydrazine with formaldehyde was dark brown colour in which DMF was used as a reaction medium. Solubility behaviours of the terpolymer sample have been studied in various solvents. The terpolymer were found to be soluble in dimethylsulphoxide (DMSO), HCl, H_2_SO_4_ but was insoluble in other solvents like chloroform, benzene, toluene, ethanol, and ether. The solubility parameter of synthesized terpolymer is shown in Table S1 of the supporting information. Based on the analytical data, the empirical formula of the repeating unit is found to be C_16_H_22_N_4_, which is in good agreement with the calculated value of C, H, N as presented in Table S2 of the supporting information.

### 3.1 Spectral characterization

#### 3.1.1 Vibrational analysis

Vibrational studies are a highly relevant tool that aid in explicating the structural properties of a molecule and provides a visual representation of a molecules structure [28]. Vibrational studies have greatly contributed to the advancement in chemistry of polymers, catalysis and also in fast reaction dynamics [29]. Herein, selected vibrations of PPHF are studied as presented in Table 1. The 36 atoms of PPHF possess 102 modes of vibration comprising of CH_3_, CH_2_, N–H and C–H vibrations. Table 1 shows the experimental and theoretical FT-IR values of the studied compound PPHF while Fig. S1 of the supporting information shows the FT-IR plot of the studied compound.

**Table 1:** Experimental and theoretical FT-IR of the studied compound.

Modes	Experimental (cm^−1^)	Theoretical (cm^−1^)	Assignment
1		3129.53	Asymmetric stretching CH_3_
2		3051.50	Asymmetric stretching CH_3_
3		3033.53	Asymmetric stretching CH_2_
4	2921.60	2984.59	Symmetric stretching CH_2_
5		2960.54	Symmetrical stretching CH_2_
6		1271.74	Wagging CH_2_
7		3629.79	Symmetric N–H
8	3304.12	3607.17	Symmetric N–H
9		3579.89	Symmetric N–H
10		3450.23	Symmetric N–H
11	3027.32	3211.72	Symmetric C–H
12		390.74	N–H

#### 3.1.2 N–H vibrations

The N–H stretching vibration is observed theoretically within the range 3629.79 cm-1 -3450.23 cm-1. The experimental range is observed at 3304.12 cm^−1^ which falls within the expected range of 3500–3200 cm^−1^ for N–H stretching vibration [30]. Also, a wagging vibration of 390.74 cm^−1^ was observed at a frequency of 390.74 cm^−1^.

#### 3.1.3 CH_3_, CH, and CH_2_ vibrations

The CH_2_ theoretical stretching vibration occurs at a frequency of 2984.59–2960.54 cm^−1^ while the experimental occurs as a frequency of 2921.60 cm^−1^. These obtained values fall within the expected range of 3000–2850 cm^−1^ [31]. An asymmetric stretching CH_2_ vibration is observed at a frequency of 3033.53 cm^−1^ and a wagging vibration is observed at a frequency of 1271.74 cm^−1^. The CH_3_ vibrations is observed theoretically at a frequency of 3129.53–3051.50 cm^−1^ while the expected range is within 3148 and 3142 cm^−1^ [32]. The experimental CH_3_ vibration is observed at 3137.35 cm^−1^. The C–H stretching vibration is expected in the range 3100–2600 cm^− 1^ [33]. The experimental C–H vibration is in agreement with this range as it is observed at a frequency of 3027.32 cm^−1^. The theoretical vibration seems to be slightly deviated as it occurs at a frequency of 3211.72 cm^−1^.

#### 3.1.4 NMR spectral studies

^1^H NMR

The NMR was scanned at 400 MHz with dimethylsulphoxide (DMSO) solvent. The signals appeared at 6.6–7.6 (*δ*) ppm is assigned to all the protons of aromatic ring. The signal showed at 10.4 (*δ*) ppm is assigned to the –NH bridge in the terpolymeric ligand. The signal observed at 4.8 (*δ*) ppm is assigned to the Ar-N-CH_2_ linkages. The signal appeared at 2.5 (*δ*) ppm is assigned to the methylene group in the terpolymer as shown in Table 2.

^13^C NMR

**Table 2:** ^1^H NMR spectral data of PPHF–I terpolymer.

Nature of the Assigned in the spectrum	Expected chemical shift(*δ*) ppm	Observed chemical shift (*δ*) ppm
Aromatic Proton (Ar–H)	6.5–8.0	6.6–7.6
Protons of –NH bridge	8.5–10.5	10.4
Methylene (-CH_2_) group	1.0–2.6	2.5
Ar-N-CH_2_	4.5–6.0	4.8

The ^13^C NMR spectrum provides useful information about the nature of the carbon skeleton present in the resin. The spectrum shows the corresponding peaks at 118.81, 112.36, 113.98, 119.17, 115.16 and 115.89 (*δ*) ppm with respect to C_1_–C_6_ of the aromatic ring of *p*-phenylenediamine. The peak appeared at 129.41, 128.80, 119.37, 129.28, 129.98 and 137.33 (*δ*) ppm, is assigned to the C_1_–C_6_ of the aromatic ring of phenyl hydrazine. The peak appeared at 40.58 (*δ*) ppm is assigned to the –CH_2_ group in the terpolymer. The peak appeared at 145.23 (*δ*) ppm is due to the C–N group as presented in Table 3.

**Table 3:** ^13^C NMR spectral data of PPHF–I.

Compound	Chemical Shift (*δ*) ppm		
	Aromatic ring (C_1_ – C_6_)	Methylene –CH_2_ in terpolymer	(C–N) group
**PPHF–I**	***p*-Phenylenediamine** 118.81, 112.36, 113.98, 119.17, 115.16,115.89 **Phenyl hydrazine** 129.41, 128.80, 119.37, 129.28, 129.98, 137.33	40.58	145.23

### 3.2 Reactivity analysis

**Fig. 1:**
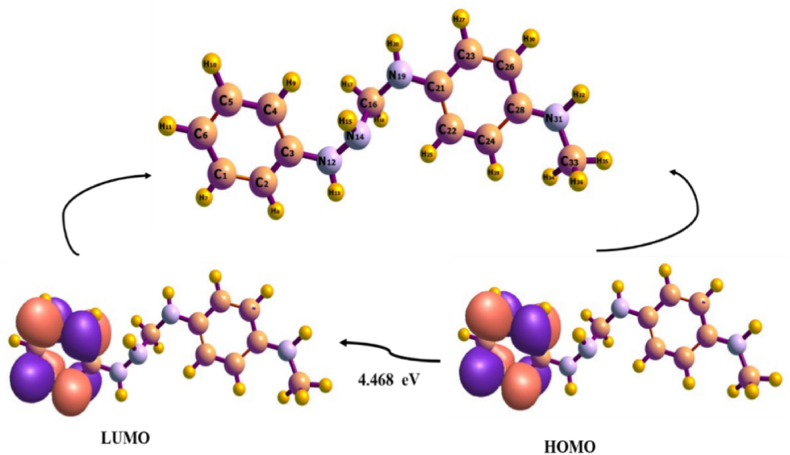
Studied structure and the HOMO LUMO plot using Chemcraft.

The Lowest Unoccupied Molecular Orbital (LUMO) and the Highest Occupied Molecular Orbital (HOMO) are the main topics of the Frontier molecular orbital (FMO) analysis [[34], [35], [36]]. Although the LUMO symbolizes the electron acceptor, the HOMO stands in for the electron donor [37]. The electric, optical, and physical properties of the molecular system, as well as its chemical reactivity and kinetic stability behaviour, are qualitatively predicted using FMO [38]. The shapes of frontier molecular orbitals, however, indicate the potential location for intra- or intermolecular interaction (or for reaction), as well as the spectroscopic characteristics of p-conjugated molecular systems, because the interactions between the HOMO and LUMO, such as UV-V is. Spectral properties, can be explained by using the molecular orbital theory [39,40]. The energy gap E_g_, which represents the disparity between HOMO and LUMO energy, is an important statistic [41]. The frontier molecular orbitals make substantial predictions about a molecule's polarizability and chemical reactivity [42]. A variety of chemical reactions that involve in the transfer of electron density from the HOMO to the LUMO are made easier by a decrease in the HOMO-LUMO gap as the system approaches a transition state [43]. The HOMO LUMO gap is a reflection of the molecule's chemical activity. The distributions of the HOMO and LUMO orbitals for the molecule mentioned in the title are shown in Fig. 1 as calculated at the same theoretical level.

Based on this study, the HOMO and LUMO are centralized on the hexane ring of the compound. The HOMO was found to be −4.786 eV, whereas the LUMO was found to be −0.318 eV. It is also noteworthy that the elevated HOMO value has a major impact on the bioactivity of the titled compound. The HOMO-LUMO energy gap provides an explanation for the charge transfer interaction that eventually takes place within the molecule (E_g_). The investigated chemical has a low energy gap of 4.468 eV and exhibits excellent reactivity and polarizability. The global chemical reactivity descriptors of molecules, such as hardness, softness, electronic chemical potential, Mulliken electronegativity, and electrophilicity index, can be described using the HOMO and LUMO energies of the molecule and the obtained values for the above mentioned is displayed in Table 4. The chemical softness, which may be computed from the opposite of hardness, is a molecule's capacity to accept electrons. The more reactivity and biological activity of the investigated substance, the higher the softness value. For the compound under study, a softness value of 0.224eV is noted. Chemical systems' resistance to changes in electron cloud density are gauged by their chemical hardness, which is perceived as the opposite of chemical softness. A low chemical hardness value of 2.234 eV is obtained. Hence, it supports the reactivity of the studied compound. The ability of an atom to draw a shared pair of electrons (or electron density) toward itself is known as electronegativity, i.e., a chemical attribute. For the investigated compound, a high electronegativity value of 2.552 eV is attained. It can be inferred from this that the compound has the capacity to draw electrons to itself, facilitating reactivity.

**Table 4:** HOMO LUMO and chemical descriptors of studied compound calculated at B3LYP-D3BJ/def2svp method.

Parameters	Value (eV)
HOMO	−4.786
LUMO	−0.318
Ionization potential	4.786
Electron affinity	0.318
Energy gap	4.468
Chemical hardness	2.234
Chemical softness	0.224
Electronegativity	2.552
Chemical potential	−2.552
Electrophilicity index	1.458

### 3.3 Natural bond orbital (NBO) analysis

The Natural Bond Orbital (NBO) study provides a wealth of knowledge regarding inter- or intramolecular interactions, which are essential to comprehending chemical processes like hydrogen bonding and conjugative interactions in molecular systems [44]. The Second Order Fock Matrix in the NBO basis was employed to evaluate the donor-acceptor interaction leading to the electron delocalization from the occupied NBO to the unoccupied NBO [45,46]. Weinhold and colleagues have provided the NBO and the concept of the Natural Atomic Orbital (NAO) analysis to derive the NBOs, occupancies, energies, and delocalization interactions [47]. The one-electron density matrix is used to define the shape of the atomic orbitals in the molecular orbital environment [48]. The knowledge of NBO method on interactions in filled and virtual orbital spaces, which could improve the investigation of intra- and intermolecular interactions, is a useful characteristic [49]. The delocalization of electron density between occupied Lewis-type (bond or lone pair) NBO orbitals and formally vacant non-Lewis (antibond or Rydberg) NBO orbitals leads to a stabilizing donor-acceptor interaction [50]. For each donor I and acceptor (j), the delocalization i/j is related to the stabilizing energy (E^2^) (j) [48]. A larger E^(2)^ denotes a stronger interaction or an increased propensity for electron donors to donate to electron acceptors as a result of electron delocalization, which increases the degree of system conjugation [49]. From the results presented in Table 5, the studies structure is stabilized by a bonding π-antibonding π* interaction due the high E^2^ values obtained for the π to π* C_1_–C_6_/C_2_–C_3_, π C_1_ - C_6_/π C_1_ - C_6_, π C_2_ - C_3_/π*C_1_ - C_6_, πC_2_ - C_3_/π*C_4_ - C_5_ with energies 19.96 kcal/mol, 20.95 kcal/mol, 20.74 kcal/mol and 20.09 kcal/mol respectively. Furthermore, this interaction is a forward donation of electron hence the greater energy observed. The total stabilization energy of 123.56kkcal/mol is observed for the studied compound. Another key interaction that contributes to the stabilization of this compound is the LP (1) - σ * interaction between LP (1) N31/σ *C26 – C28, LP (1) N14/σ *C16 – H17 with total stabilization energy of 18.22 kcal/mol. Other interactions that contribute to the stability include the LP (1) – π* with total stabilization energy of 8.16 kcal/mol and σ - σ* with total stabilization energy of 18.03 kcal/mol. Other interactions and their contribution to the stability of the studied compound are found in Table 5.

**Table 5:** Natural bond orbital (NBO) analysis calculated at B3LYP-D3BJ/def2svp method.

Donor	Acceptor	E^2^ kcal/mol	E(i)-E(j)	F (i, j)
π C1 – C6	π*C2 – C3	19.96	0.28	0.068
π C1 – C6	π*C4 – C5	20.95	0.28	0.069
π C2 – C3	π*C1 – C6	20.74	0.29	0.069
πC2 - C3	π*C4 – C5	20.09	0.29	0.068
π C4 – C5	π*C1 – C6	20.12	0.28	0.068
πC4 - C5	π*C2 – C3	21.70	0.29	0.071
		**123.56**		
LP (1) N31	σ *C26 – C28	5.63	0.86	0.063
LP (1) N31	σ *C33 – H36	6.39	0.77	0.064
LP (1) N14	σ *C16 – H17	6.20	0.80	0.063
		**18.22**		
LP (1) N12	π*C2 – C3	3.69	0.34	0.034
LP (1) N31	π*C26 – C28	4.47	0.32	0.036
		**8.16**		
σ N31 – H32	σ C24 – C28	3.02	1.17	0.053
σ N31 – H32	σ *C33 – H34	2.37	1.09	0.045
σ C28 – N31	σ *C33 – H35	1.55	1.20	0.039
σ C26 – H30	σ *C24 – C28	4.41	1.09	0.062
σ C26 – H30	σ *C21 – C23	3.61	1.09	0.056
σ C26 – H30	σ *C23 – C26	1.05	1.09	0.030
σ C28 – N31	σ *C22 – C24	2.02	1.27	0.045
		**18.03**		
πC26 - C28	σ *N31 – C33	1.47	0.61	0.029

### 3.4 Molecular electrostatic potential

**Fig. 2:**
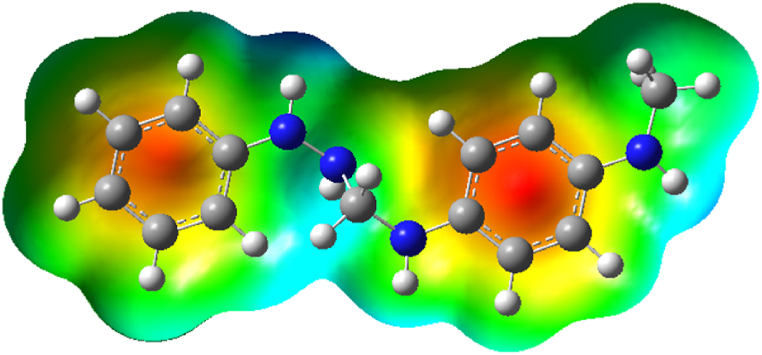
Molecular electrostatic map of PPHF.

It has become very important to map molecular electrostatic potential (MESP) maps for chemical compounds because it could reveal important details about the interaction, active sites, and the type of chemical addition that a molecule is most likely to experience either an electrophilic attack or a nucleophilic attack as well as other pertinent information [51]. The concept of MESP is useful in molecular modeling calculations since it can provide fairly accurate data on the active sites of various chemical entities [52]. Also, it is important to determine whether a chemical structure will most likely undergo electrophilic or nucleophilic chemical addition when examining the type of chemical addition. There are different definition states for the molecular surface. Some scientists think of MESP maps as the outer region of a structure made up of intersecting spheres whose centres line up with the nuclei of all atoms [53]. These spheres are created using the interested atoms' van der Waals radii. Nevertheless, Bader and colleagues defined MESP as the exterior contour of an object's electron density. The extreme negative and positive sites, respectively, in the target structure are indicated by various colour in the generated MESP maps, which range from red to dark blue. Red, yellow, green, light blue, and dark blue are the colour that make up the entire map, going from most negative to most positive. Dark blue colour represents extremely positive potentials, whereas red colour represents extremely negative potentials [56]. In comparison to red, the possible negative zone is reduced in the yellow colour. Similar to how dark blue regions have more positive potentials than light blue ones, so do light blue ones as display in Fig. 2. When compared to red and dark blue potentials, green represents areas that are almost neutral. The relationship between the electronegativity of the bound atoms can be used to relate the distribution of potentials and colours in some way. When coupled with less electronegative atoms, highly electronegative atoms will turn red. The colour distribution is substantially narrower when the electronegative values of the atoms are close together. From the studied compound PPHF, an electron cloud localized in the middle of the benzene rings, which appears as a red colour with yellow extreme in the centre, is what characterizes the shown MESP. The electron delocalization and electron resonance feature found in each benzene ring may be responsible for this cloud. Intense blue colour is observed within the Nitrogen and hydrogen atom which indicates a positive region and tis is attributed to the electronegative nature of nitrogen. The light blue coloration observed at the hydrogen atom the light blue coloration may be due to the hydrogen atoms scattered virtually all round the studied compound. Then, green colour is observed which signifies neutral region.

### 3.5 Molecular docking analysis

A computer method called molecular docking is used to forecast the mechanism and affinities of a ligand's (a tiny molecule's) binding to a target protein [[54], [55], [56]]. This is achieved through series of procedures which include: protein preparation involving addition of polar hydrogen atoms, allocating charges, and eliminating any potential ligands like water molecules [[57], [58], [59], [60]], improving the ligand structures so as to reduce its energy and guarantee its stability. Docking the ligand to the protein active site using the docking software which provides a wide range of orientation and conformation for the ligand in the binding site. Finally, examining the docking pose with the highest binding affinity and higher number of hydrogen bonds [61]. In order to forecast the binding affinities and binding modes, a wide variety of software, can be used in forecasting the binding mode and affinities of a ligand with targeted protein such software include Auto dock, Glide, Dock and Gold. Essentially, the molecular docking approach incorporates a series of computational and experimental processes to anticipate the binding mechanism and affinity of a ligand with a target protein. It can offer insights into the binding mechanism and aid in the optimization of the design of novel drug candidates with increased potency and selectivity, making it a useful tool in drug discovery and design [59,61]. Quantum mechanics computations alongside molecular dynamics simulations can be integrated with molecular docking to provide a more thorough knowledge of protein-ligand interactions and binding mechanisms [60,]. Protein-ligand interactions in a variety of situations, such as enzyme inhibition, protein-protein interactions, and receptor-ligand interactions, can be studied using molecular docking [59]. The quality of the protein structure, the choice of docking software and parameters, and the experimental validation of the results all affect how accurately molecular docking predictions turn out [59].

**Fig. 3:**
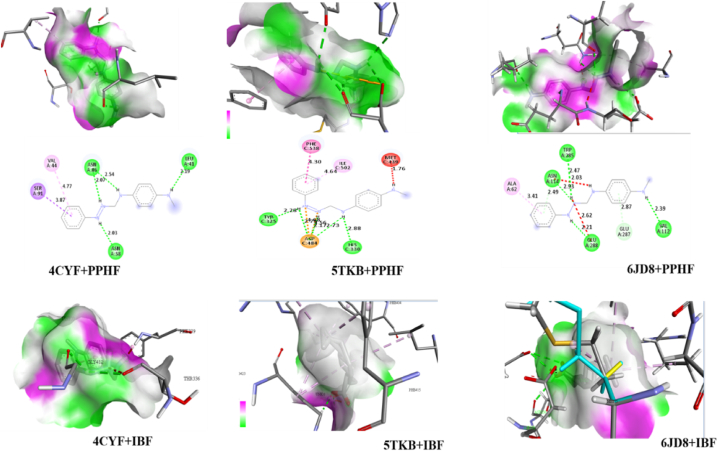
The Biovia visualization unveiling the intricate hydrogen bonding network, complemented by a compelling 2D representation, showcasing the dynamic interaction between the proteins (4CYF, 5TKB, and 6JDB), the ligand (PPHF), and the standard drug (IBF).

**Fig. 4:**
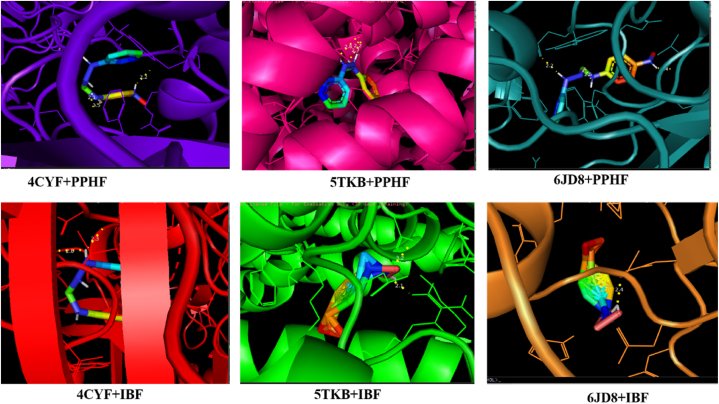
PyMol visualization illustrating the favorable 3D interactions between the proteins (4CYF, 5TKB, and 6JDB) and the ligand (PPHF), along with the standard drug (IBF) interactions.

From the obtained result, the studied compound has a good biological activity with anti-inflammatory proteins 6JD8, 5TKB and 4CYF as seen in Table 6. On interaction of PPHF and 6JD8, a binding affinity of −4.5 kcal/mol was observed, affinity of −7.8 kcal/mol was observed on interaction with 5TKB while a bonding affinity of −8.1 kcal/mol was observed for 4CYF. The obtained binding affinities of the studied compound with proteins, comparable to those observed with the standard drug ibuprofen (IBF) at −5.9 kcal/mol, −7.0 kcal/mol, and −6.1 kcal/mol respectively, suggest that the studied compound exhibits similar potential for interaction and biological activity. This similarity may be attributed, at least in part, to the presence of nitrogen, a heteroatom within the studied compound. In terms of hydrogen bonding, it is known that a higher number of hydrogen bond interactions contributes to increased biological reactivity of a compound. In the case of interacting PPHF with proteins 6JD8, 5TKW, and 4CYF, 4, 5, and 4 hydrogen bond interactions were respectively observed, indicating a significant potential for biological reactivity and interaction between the studied compound and these proteins. The highest number of hydrogen bond is observed on the interaction with 5TKB. When the standard drug was interacted with the proteins, 4, 2 and 4 hydrogen bond interactions for 6JD8, 5TKW and 4CYF was observed. The bond distance also plays a vital role in determining the extent of biological potency of a compound. The shorter the bond distance the more reactive the molecule is and vice versa. The bond distance of the various interaction is shown in Table 6. The amino acid residue involved in this study include 6JD8: A:ASN:114, A:TRP:285, A:GLU:288, A:VAL:112, A:ASP:256, A:ASP:233, A:ARG:72 and A:MET:72. 5TKW: C:TYR:325, C:HIS:330, C:ASP:484, A:ARG:42 and B:ASN:38. The amino acid residue involved on interaction with 4CYF are A: ASN: 86 and A: LEU: 41. The amino acid residue involved in the interaction of IBF and 6JD8 are A:ASP:256, A:ASP:233, A:ARG:72, A:MET:72, while on interaction with A:ARG:42, B:ASN:38 and on interaction with 4CYF, A:GLY:142 and A:THR:336 were obtained. Fig. 3, Fig. 4 shows the 2D and 3D visualization of the protein ligand interaction.

**Table 6:** Binding affinities, hydrogen bonds, amino acid residues and bond distances of protein-ligand interaction.

Interaction	Best pose (kcal/mol)	No of hydrogen bonds	Amino acid residue	Bond distance
6JD8+PPHF	−4.9	4	A:ASN:114	2.49
			A:TRP:285	2.93
			A:GLU:288	2.21
			A:VAL:112	2.39
6JD8+1BF	−5.9	4	A:ASP:256	2.92
			A:ASP:233	2.79
			A:ARG:72	2.96
			A:MET:72	3.33
5TKB + PPHF	−7.8	5	C:TYR:325	2.28
			C:HIS:330	2.88
			C:ASP:484	2.73
			C:ASP:484	2.17
			C:ASP:484	2.75
5TKB + IBF	−7.0	2	A:ARG:42	2.12
			B:ASN:38	3.24
4CYF + PPHF	−8.1	4	A:ASN:86	2.07
			A:ASN:86	2.54
			A:ASN:38	2.03
			A:LEU:41	2.19
4CYF + IBF	−6.1	2	A:GLY:142	2.82
			A:THR:336	2.28

## 4 Conclusions

In this present study, the biological potency of *p*-phenylenediamine-phenylhydrazine-formaldehyde (PPHF) as an anti-inflammatory agent is studied. The theoretical calculations were carried out using the Density function theory at the B3LYP/6–31 G (d, p) level of theory. The experimental and theoretical frequencies were compared through the FT-IR analysis. The Frontier molecular alongside global quantum descriptors were calculated. In order to further elucidate on the charge transfer and stability of the studied compound, the Natural bond orbital analysis based on the second order perturbation theory was analyzed and the MESP map was also obtained so as to elucidate areas of nucleophilic and electrophilic attack. Furthermore, to provide an insight on the biological potency of the studied compound, molecular docking was also carried out against three inflammatory protein 6JD8, 4CYF and 5TKB and a commercial drug ibuprofen. The key vibration modes obtained are the C–H, CH_2_, CH_3_ and the N–H vibration. An energy gap of 4.468 eV was obtained from the studied compound suggesting biological reactivity and polarity of the studied compound. The NBo analysis shows that the stability of the studied compound is due to mainly the π to π* interaction though other interactions contribute to the stability of the compound. The MESP analysis shows the presence of a red colour with yellow ends in the midst of the benzene ring which signifies high electron delocalization within the benzene rings. The molecular docking result further confirm the biological activity of the studied compound with binding affinities of −4.9, −7.8 and −8.1 kcal/mol for 6JD8, 5TKB and 4CYF respectively. These values are comparable with those obtained for the commercial drug ibuprofen which is used in the treatment of inflammatory disease. Hence, we recommend *p*-phenylenediamine-phenylhydrazine-formaldehyde (PPHF for further studies so as to fully explore its potential as an anti-inflammatory agent.

## Author contribution statement

N. Mujafarkani, Hitler Louis: Conceived and designed the experiments; Contributed reagents, materials, analysis tools or data.

Victoria Bassey, Jumbo J. Tokono, A. Jafar Ahamed: Performed the experiments.

Innocent Benjamin, Daniel C. Agurokpon, Yohanna J. Waliya: wrote the paper. analyzed and interpreted the data.

## 5 Data availability statement

Data will be made available on request.

## Funding

This research did not receive funding from any source.

## Availability of data

All data are available within the manuscript and the manuscript’s supporting information.

## Declaration of competing interest

The authors declare that they have no known competing financial interests or personal relationships that could have appeared to influence the work reported in this paper.

## References

[1] Iacopino A.M. (2001). Periodontitis and diabetes interrelationships: role of inflammation. Ann. Periodontol..

[2] Dobson G.P., Biros E., Letson H.L., Morris J.L. (2021). Living in a hostile world: inflammation, new drug development, and coronavirus. Front. Immunol..

[3] Mayer L., Bhikha R. (2013).

[4] Garg T., Rath G., Goyal A. (2014). Ancient and advanced approaches for the treatment of an inflammatory autoimmune disease− psoriasis. Crit. Rev. Ther. Drug Carrier Syst..

[5] Manokaran S., Souza J.N.D., Manogna S., Philip S., Gupta P.K., Ah M.R. (2021). A comprehensive review on the antigen specific therapy for multiple sclerosis. SPAST Abstracts.

[6] Bradley J. (2008). TNF‐mediated inflammatory disease. J. Pathol.: J. Pathol. Society Great Britain Ireland.

[7] Ng S.C., Bernstein C.N., Vatn M.H., Lakatos P.L., Loftus E.V., Tysk C., Colombel J.F. (2013). Geographical variability and environmental risk factors in inflammatory bowel disease. Gut.

[8] Placha D., Jampilek J. (2021). Chronic inflammatory diseases, anti-inflammatory agents and their delivery nanosystems. Pharmaceutics.

[9] Jones J.L., Nguyen G.C., Benchimol E.I., Bernstein C.N., Bitton A., Kaplan G.G., Otley A.R. (2019). The impact of inflammatory bowel disease in Canada 2018: quality of life. J. Canadian Associat. Gastroenterol..

[10] Benchimol E.I., Mack D.R., Guttmann A., Nguyen G.C., To T., Mojaverian N., Manuel D.G. (2015). Inflammatory bowel disease in immigrants to Canada and their children: a population-based cohort study. Off. J. Am. College Gastroenterol.| ACG.

[11] Gisbert J.P., Chaparro M. (2014). Systematic review with meta‐analysis: inflammatory bowel disease in the elderly. Aliment. Pharmacol. Ther..

[12] Bosma-den Boer M.M., van Wetten M.L., Pruimboom L. (2012). Chronic inflammatory diseases are stimulated by current lifestyle: how diet, stress levels and medication prevent our body from recovering. Nutr. Metab..

[13] Ament M.E. (1975). Inflammatory disease of the colon: ulcerative colitis and Crohn's colitis. J. Pediatr..

[14] Refat H.M., Fadda A.A. (2015). Synthesis and antimicrobial activity of some novel Hydrazide, pyrazole, triazine, Isoxazole, and pyrimidine derivatives. J. Heterocycl. Chem..

[15] Mousa T.H., Al-Obaidi Z.M.J., Alkhafaji S.L. (2021). Molecular docking studies and evaluation of the anti-inflammatory activity of ibuprofen-tranexamic acid codrug. Lat. Am. J. Pharm..

[16] Hamed M.M., El-Dean A.M.K., Abdel-Mohsen S.A., Tolba M.S. (2021). New diclofenac derivatives as anti-microbial, anti-inflammatory agents: design, synthesis, biological screening, and molecular docking study. Russ. J. Bioorg. Chem..

[17] Abdelall E.K., Lamie P.F., Ahmed A.K., EL-Shaymaa E.N. (2019). COX-1/COX-2 inhibition assays and histopathological study of the new designed anti-inflammatory agent with a pyrazolopyrimidine core. Bioorg. Chem..

[18] Anoop P. (2012).

[19] Badr M.H., Elbayaa R.Y., El-Ashmawy I.M. (2013). Design, synthesis and molecular docking study of some substituted 4, 5-dihydro-2H-indazole derivatives as potential anti-inflammatory agents. Med. Chem..

[20] El-Hawash A.M., Soliman S., Youssef R.M., Ragab M.A., As Elzahhara P., M El-Ashmawey I., A Shaat I. (2014). Design, synthesis and biological screening of some pyridinylpyrazole and pyridinylisoxazole derivatives as potential anti-inflammatory, analgesic, antipyretic and antimicrobial agents. Med. Chem..

[21] Stewart B., Hylton D.J., Ravi N. (2013). A systematic approach for understanding slater–Gaussian functions in computational chemistry. J. Chem. Educ..

[22] Trott O., Olson A.J. (2010). AutoDock Vina: improving the speed and accuracy of docking with a new scoring function, efficient optimization, and multithreading. J. Comput. Chem..

[23] Sharma S., Sharma A., Gupta U. (2021).

[24] Choudhury D., Biswas S. (2021). Structure-guided protein engineering of human cathepsin L for efficient collagenolytic activity. Protein Eng. Des. Sel..

[25] Moslin R., Gardner D., Santella J., Zhang Y., Duncia J.V., Liu C., Weinstein D.S. (2017). Identification of imidazo [1, 2-b] pyridazine TYK2 pseudokinase ligands as potent and selective allosteric inhibitors of TYK2 signalling. Medchemcomm.

[26] Boersma Y.L., Newman J., Adams T.E., Cowieson N., Krippner G., Bozaoglu K., Peat T.S. (2014). The structure of vanin 1: a key enzyme linking metabolic disease and inflammation. Acta Crystallogr., Sect. D: Biol. Crystallogr..

[27] The US National Science Foundation, National Institutes of Health, and Department of Energy (1999). https://www.rcsb.org/pages/about-us/index.

[28] Noureddine O., Gatfaoui S., Brandan S.A., Sagaama A., Marouani H., Issaoui N. (2020). Experimental and DFT studies on the molecular structure, spectroscopic properties, and molecular docking of 4-phenylpiperazine-1-ium dihydrogen phosphate. J. Mol. Struct..

[29] Deglmann P., Schäfer A., Lennartz C. (2015). Application of quantum calculations in the chemical industry—an overview. Int. J. Quant. Chem..

[30] Zabokrycka A., Klaeboe P., Cyvin B.N., Cyvin S.J., Brunvoll J. (1982). 1-Phenylazo-2-naphthylamine complex compounds: infrared and Raman spectra of 1-phenylazo-2-napthylamine and empirical assignment of ir spectra of bis (1-phenylazo-2-naphthylaminato) nickel (II) complex. Spectrochim. Acta Mol. Spectros.

[31] Trabelsi S., Issaoui N., Brandán S.A., Bardak F., Roisnel T., Atac A., Marouani H. (2019). Synthesis and physic-chemical properties of a novel chromate compound with potential biological applications, bis (2-phenylethylammonium) chromate (VI). J. Mol. Struct..

[32] Manceau M., Rivaton A., Gardette J.L., Guillerez S., Lemaître N. (2009). The mechanism of photo-and thermooxidation of poly (3-hexylthiophene)(P3HT) reconsidered. Polym. Degrad. Stabil..

[33] Chandrasekar S., Balachandran V., Evans H.S., Latha A. (2015). Synthesis, crystal structures HOMO–LUMO analysis and DFT calculation of new complexes of p-substituted dibenzyltin chlorides and 1, 10-phenanthroline. Spectrochim. Acta Mol. Biomol. Spectrosc..

[34] Jomaa I., Issaoui N., Roisnel T., Marouani H. (2021). Insight into non-covalent interactions in a tetrachlorocadmate salt with promising NLO properties: experimental and computational analysis. J. Mol. Struct..

[35] Issaoui N., Ghalla H., Bardak F., Karabacak M., Dlala N.A., Flakus H.T., Oujia B. (2017). Combined experimental and theoretical studies on the molecular structures, spectroscopy, and inhibitor activity of 3-(2-thienyl) acrylic acid through AIM, NBO, FT-IR, FT-Raman, UV and HOMO-LUMO analyses, and molecular docking. J. Mol. Struct..

[36] Ramalingam A., Sambandam S., Medimagh M., Al-Dossary O., Issaoui N., Wojcik M.J. (2021). Study of a new piperidone as an anti-Alzheimer agent: molecular docking, electronic and intermolecular interaction investigations by DFT method. J. King Saud Univ. Sci..

[37] Benjamin I., Udoikono A.D., Louis H., Agwamba E.C., Unimuke T.O., Owen A.E., Adeyinka A.S. (2022). Antimalarial potential of naphthalene-sulfonic acid derivatives: molecular electronic properties, vibrational assignments, and in-silico molecular docking studies. J. Mol. Struct..

[38] Agwamba E.C., Benjamin I., Louis H., Udoikono A.D., Igbalagh A.T., Egemonye T.C., Adeyinka A.S. (2022). Antitubercolusic potential of amino-(formylphenyl) diazenyl-hydroxyl and nitro-substituted naphthalene-sulfonic acid derivatives: experimental and theoretical investigations. Chemistry Africa.

[39] Asogwa F.C., Agwamba E.C., Louis H., Muozie M.C., Benjamin I., Gber T.E., Ikeuba A.I. (2022). Structural benchmarking, density functional theory simulation, spectroscopic investigation and molecular docking of N-(1H-pyrrol-2-yl) methylene)-4-methylaniline as castration-resistant prostate cancer chemotherapeutic agent. Chemical Physics Impact.

[40] Eno E.A., Mbonu J.I., Louis H., Patrick-Inezi F.S., Gber T.E., Unimuke T.O., Offiong O.E. (2022). Antimicrobial activities of 1-phenyl-3-methyl-4-trichloroacetyl-pyrazolone: experimental, DFT studies, and molecular docking investigation. J. Indian Chem. Soc..

[41] Eno E.A., Louis H., Ekoja P., Benjamin I., Adalikwu S.A., Orosun M.M., Agwamba E.C. (2022). Experimental and computational modeling of the biological activity of benzaldehyde sulphur trioxide as a potential drug for the treatment of Alzheimer disease. J. Indian Chem. Soc..

[42] Ntui T.N., Oyo-Ita E.E., Agwupuye J.A., Benjamin I., Eko I.J., Ubana E.I., Imojara A. (2023). Synthesis, spectroscopic, DFT study, and molecular modeling of thiophene-carbonitrile against enoyl-ACP reductase receptor. Chemistry Africa.

[43] Apebende C.G., Idante P.S., Louis H., Ameuru U.S., Unimuke T.O., Gber T.E., Asogwa F.C. (2022). Integrated spectroscopic, bio-active prediction and analytics of isoquinoline derivative for breast cancer mitigation. Chemistry Africa.

[44] Gber T.E., Louis H., Owen A.E., Etinwa B.E., Benjamin I., Asogwa F.C., Eno E.A. (2022). Heteroatoms (Si, B, N, and P) doped 2D monolayer MoS 2 for NH 3 gas detection. RSC Adv..

[45] Louis H., Charlie D.E., Amodu I.O., Benjamin I., Gber T.E., Agwamba E.C., Adeyinka A.S. (2022). Probing the reactions of thiourea (CH4N2S) with metals (X= Au, Hf, Hg, Ir, Os, W, Pt, and Re) anchored on fullerene surfaces (C59X). ACS Omega.

[46] Makhlouf J., Louis H., Benjamin I., Ukwenya E., Valkonen A., Smirani W. (2023). Single crystal investigations, spectral analysis, DFT studies, antioxidants, and molecular docking investigations of novel hexaisothiocyanato chromate complex. J. Mol. Struct..

[47] Agwamba E.C., Udoikono A.D., Louis H., Udoh E.U., Benjamin I., Igbalagh A.T., Ushaka U.B. (2022). Synthesis, characterization, DFT studies, and molecular modeling of azo dye derivatives as potential candidate for trypanosomiasis treatment. Chemical Phys. Impact.

[48] Benjamin I., Gber T.E., Louis H., Ntui T.N., Oyo-Ita E.I., Unimuke T.O., Adeyinka A.S. (2022). Modelling of aminothiophene-carbonitrile derivatives as potential drug candidates for hepatitis B and C. Iran. J. Sci. Technol. Trans. A-Science.

[49] Inah B.E., Louis H., Benjamin I., Unimuke T.O., Adeyinka A.S. (2022). Computational study on the interactions of functionalized C24NC (NC= C,–OH,–NH2,–COOH, and B) with chloroethylphenylbutanoic acid. Can. J. Chem..

[50] Edet H.O., Louis H., Gber T.E., Idante P.S., Egemonye T.C., Ashishie P.B., Adeyinka A.S. (2023). Heteroatoms (B, N, S) doped quantum dots as potential drug delivery system for isoniazid: insight from DFT, NCI, and QTAIM. Heliyon.

[51] Lakshminarayanan S., Jeyasingh V., Murugesan K., Selvapalam N., Dass G. (2021). Molecular electrostatic potential (MEP) surface analysis of chemo sensors: an extra supporting hand for strength, selectivity & non-traditional interactions. J. Photochem. Photobiol., A.

[52] Daolio A., Pizzi A., Calabrese M., Terraneo G., Bordignon S., Frontera A., Resnati G. (2021). Molecular electrostatic potential and noncovalent interactions in derivatives of group 8 elements. Angew. Chem..

[53] Hassan A.S. (2022). Antimicrobial evaluation, in silico ADMET prediction, molecular docking, and molecular electrostatic potential of pyrazole-isatin and pyrazole-indole hybrid molecules. J. Iran. Chem. Soc..

[54] Towseef Ahmad H., Mohammed Ameen K.K., Saleem H., Syed Ali Padusha M., Mashood Ahamed F.M. (2023). Molecular structure determination, spectroscopic, quantum computational studies and molecular docking of 4-(E)-[2-(benzylamino) phenylimino) methyl-2] ethoxy phenol. J. Biomol. Struct. Dyn..

[55] Sagaama A., Issaoui N., Al-Dossary O., Kazachenko A.S., Wojcik M.J. (2021). Non covalent interactions and molecular docking studies on morphine compound. J. King Saud Univ. Sci..

[56] Al-Zaqri N., Pooventhiran T., Rao D.J., Alsalme A., Warad I., Thomas R. (2021). Structure, conformational dynamics, quantum mechanical studies and potential biological activity analysis of multiple sclerosis medicine ozanimod. J. Mol. Struct..

[57] Thadathil D.A., Varghese S., Akshaya K.B., Thomas R., Varghese A. (2019). An insight into photophysical investigation of (E)-2-Fluoro-N’-(1-(4-Nitrophenyl) ethylidene) benzohydrazide through solvatochromism approaches and computational studies. J. Fluoresc..

[58] Elangovan N., Thomas R., Sowrirajan S., Irfan A. (2021). Synthesis, spectral and quantum mechanical studies and molecular docking studies of Schiff base (E) 2-hydroxy-5-(((4-(N-pyrimidin-2-yl) sulfamoyl) phenyl) imino) methyl benzoic acid from 5-formyl salicylic acid and sulfadiazine. J. Indian Chem. Soc..

[59] Hajam T.A., Mashood Ahamed F.M. (2022). Structural, vibrational spectroscopy, molecular docking, DFT studies and antibacterial activity of (E)-N1-(3-chlorobenzylidene) benzene-1, 4-diamine. J. Biomol. Struct. Dyn..

[60] Surendar P., Pooventhiran T., Rajam S., Bhattacharyya U., Bakht M.A., Thomas R. (2021). Quasi liquid Schiff bases from trans-2-hexenal and cytosine and l-leucine with potential antieczematic and antiarthritic activities: synthesis, structure and quantum mechanical studies. J. Mol. Liq..

[61] Ahamed F.M., Chinnam S., Challa M., Kariyanna G., Kumer A., Jadoun S., Salawi A., Al-Sehemi A G., Chakma U., Mashud M.A., Kumari I. (2023). Molecular dynamics simulation, QSAR, DFT, molecular docking, ADMET, and synthesis of ethyl 3-((5-Bromopyridin-2-yl) imino) butanoate Analogues as potential inhibitors of SARS-CoV-2. Polycycl. Aromat. Comp..

