# Supplementary material for: Synthesis, characterization, and molecular modeling of phenylenediamine-phenylhydrazine-formaldehyde terpolymer (PPHF) as potent anti-inflammatory agent
Source: Heliyon. 2023 Jul 6;9(7):e18067. doi: 10.1016/j.heliyon.2023.e18067 (PMC10362139; doi:10.1016/j.heliyon.2023.e18067)
Supplement: Multimedia component 1 [file mmc1.docx]

**Supporting information**

**Synthesis, characterization, and molecular modeling of phenylenediamine-phenylhydrazine-formaldehyde terpolymer (PPHF) as potent inflammatory agent**

**N. Mujafarkani ^1^, Victoria Bassey ^2,3^, Jumbo J. Tokono ^2,3^, A. Jafar Ahamed ^1^, Innocent Benjamin ^2^ **, Daniel C. Agurokpon ^2, 4^, Yohanna J. Waliya ^2^, Hitler Louis ^2, 3, 5**^**

*^1^PG and Research Department of Chemistry, Jamal Mohamed College (Autonomous),*

*(Affiliated to Bharathidasan University), Tiruchirappalli-620020, Tamilnadu, India*

^2^ Computational and Bio-Simulation Research Group, University of Calabar, Calabar, Nigeria

^3^ Department of Pure and Applied Chemistry, University of Calabar, Calabar, Nigeria

^4^Department of Microbiology, University of Cross River State, Calabar

*^5^Faculty of Allied Health Sciences, Chettinad Hospital and Research Institute, Chettinad Academy of Research and Education, Kelambakkam 603103, Tamil Nadu, India.*

**^**^Corresponding author’s email:** [louismuzong@gmail.com and](mailto:louismuzong@gmail.com%20and) benjamininnocent53@gmail.com

**Table S1:** Average molecular weight of PPHF-I Terpolymer

| **Sample** | **__**  **M_w_** | **__**  **M_n_** | **Polydispersity**  **(M_w_ / M_n_)** |
| --- | --- | --- | --- |
| PPHF | 1370 | 1325 | 1.0339 |

**Table S2:** Elemental analysis of PPHF- I Terpolymer

| **Terpolymer**  **Resin** | **Empirical**  **Formula of the repeating units** | **Formula mass of the repeating unit** | **Elemental Analysis (%)** | | | |
| --- | --- | --- | --- | --- | --- | --- |
|  |  |  | **C**  **(Calc.)** | **H**  **(Calc.)** | **N**  **(Calc.)** | **Cu**  **(Calc.)** |
| PPHF | C_16_H_22_N_4_ | 270.38 | 71.11  (71.23) | 8.14  (8.21) | 5.18  (5.25) | - |
| PPHF-Cu | C_32_H_44_N_8_Cu^2+^ | 603.29 | 63.68  (63.75) | 7.29  (7.37) | 18.57  (18.69) | 10.20  (10.31) |


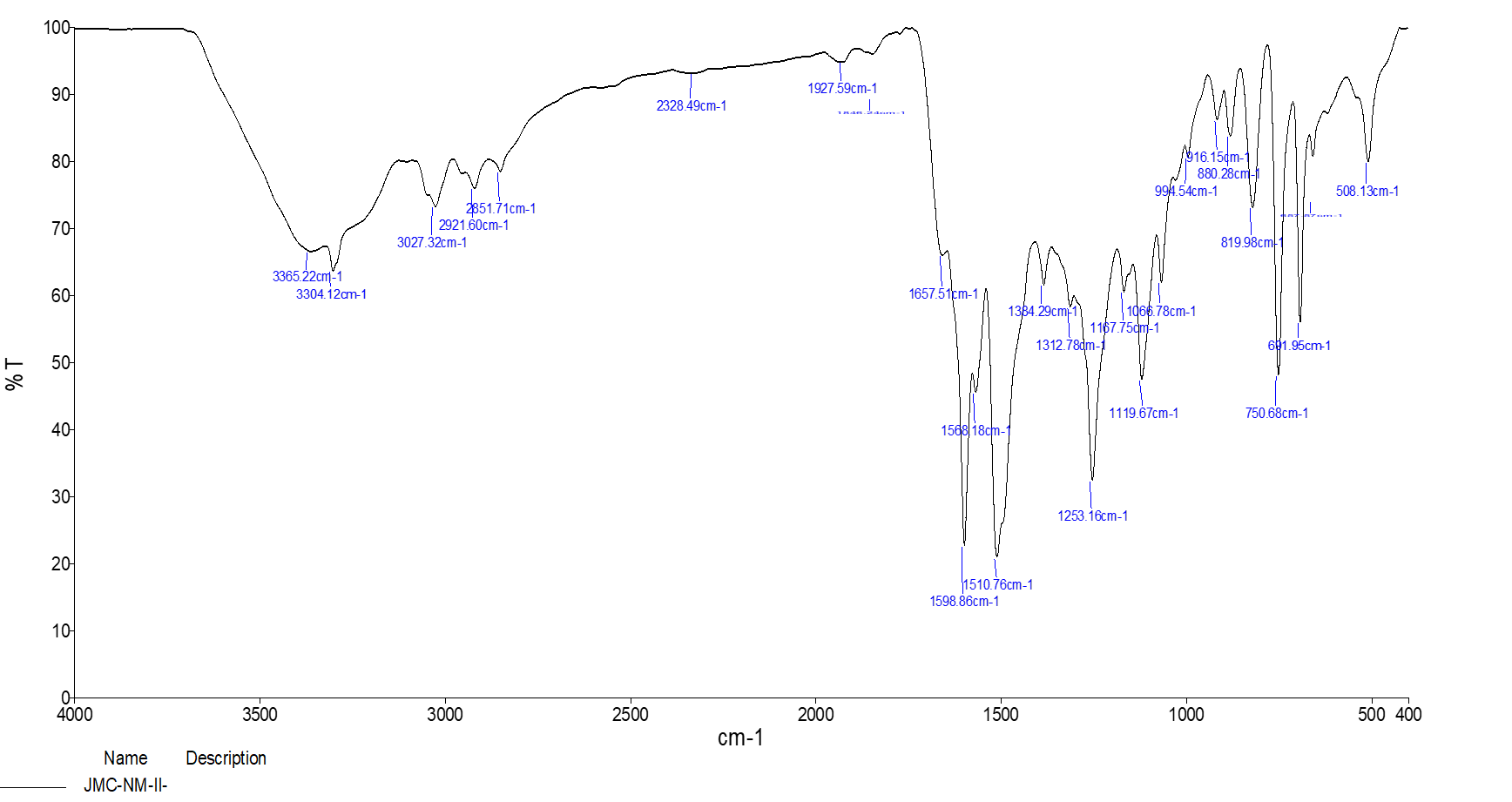


**Figure S1: FT-IR of PPHF**


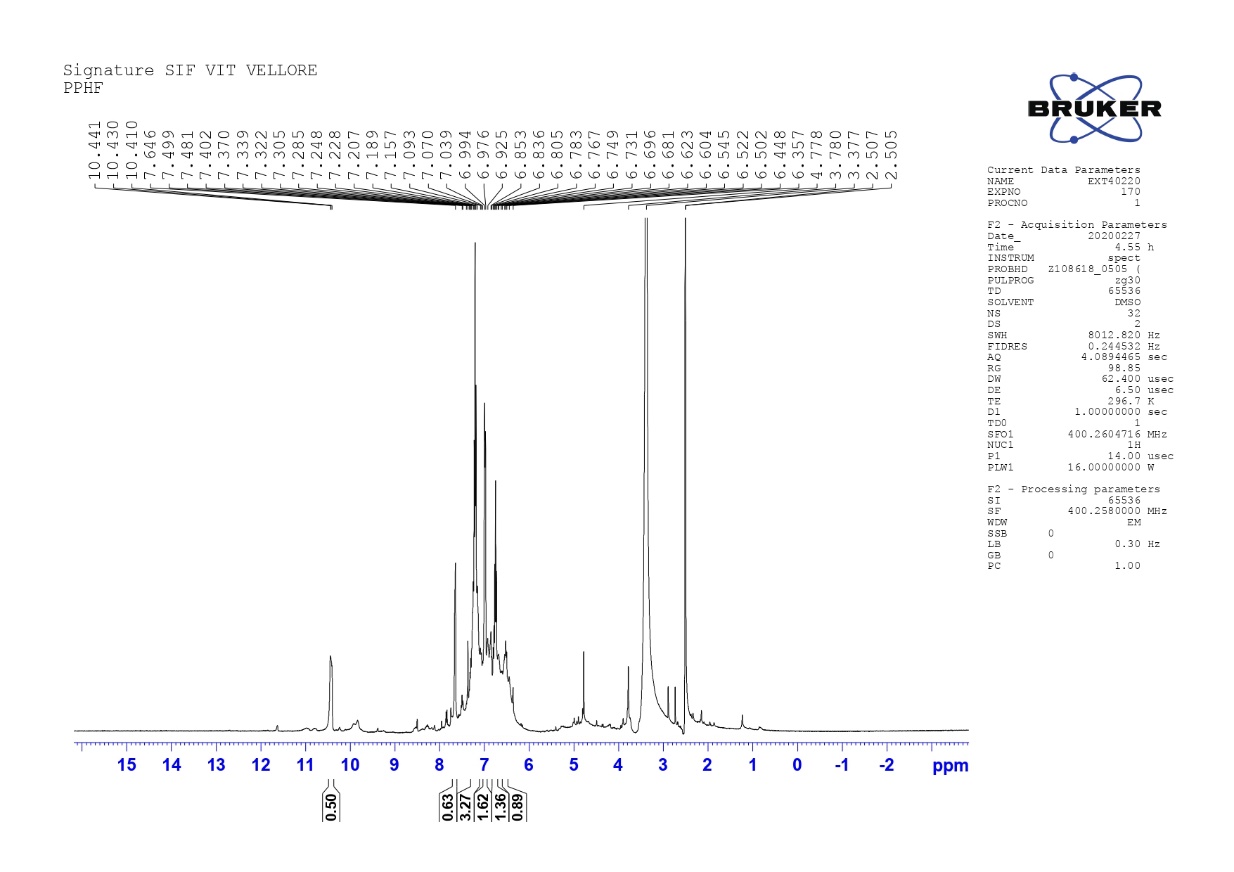


**Figure S2: H-NMR of PPHF**


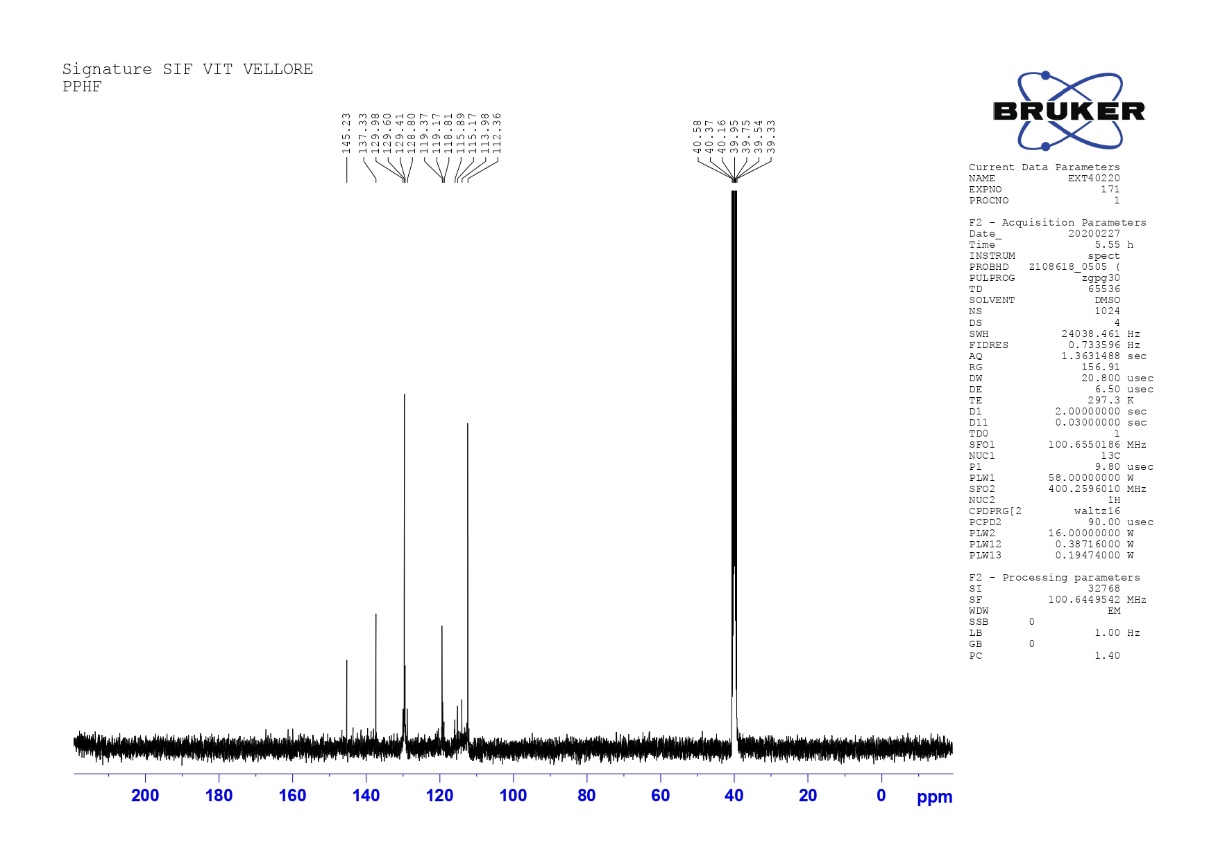


^13^C NMR spectrum of PPHF-I Terpolymer
